# Circular RNA hsa_circ_0004689 (circSWT1) promotes NSCLC progression via the miR‐370‐3p/SNAIL axis by inducing cell epithelial‐mesenchymal transition (EMT)

**DOI:** 10.1002/cam4.5527

**Published:** 2022-12-19

**Authors:** Xiang Long, Ding‐Guo Wang, Zhi‐Bo Wu, Zhong‐Min Liao, Jian‐Jun Xu

**Affiliations:** ^1^ Department of Cardiothoracic Surgery The Second Affiliated Hospital of Nanchang University Nanchang People's Republic of China

**Keywords:** circSWT1, EMT, invasion and metastasis, NSCLC, SNAIL

## Abstract

**Background:**

Previous studies have reported the role of circular RNAs (circRNAs) in the progression of non‐small‐cell lung cancer (NSCLC). SWT1‐derived circRNAs were confirmed to affect the apoptosis of cardiomyocytes; however, the biological functions of SWT1‐derived circRNAs in cancers are still unknown. Here, we investigated the potential role of SWT1‐derived circRNAs in NSCLC.

**Methods:**

We used quantitative real‐time polymerase chain reaction (qRT‐PCR) to measure the expression of circSWT1 in NSCLC tissues and paired normal tissues. The potential functions of circSWT1 in tumor progression were assessed by CCK‐8, colony formation, wound healing, and matrigel transwell assays in vitro and by xenograft tumor models in vivo. Next, epithelial‐mesenchymal transition (EMT) was evaluated by western blotting, immunofluorescence, and immunohistochemistry (IHC). Moreover, circRIP, RNA pulldown assays, luciferase reporter gene assays, and FISH were conducted to illuminate the molecular mechanisms of circSWT1 via the miR‐370‐3p/SNAIL signal pathway. Then, we knocked out SNAIL in A549 and H1299 cells to identify the roles of circSWT1 in the progression and EMT of NSCLC through SNAIL. Finally, circSWT1 functions were confirmed in vivo using xenograft tumor models.

**Results:**

CircSWT1 expression was significantly upregulated in NSCLC tissues, and high expression of circSWT1 predicted poor prognosis in NSCLC via survival analysis. In addition, overexpression of circSWT1 promoted the invasion and migration of NSCLC cells. Subsequently, we found that overexpression of circSWT1 induced EMT and that knockdown of circSWT1 inhibited EMT in NSCLC cells. Mechanistically, circSWT1 relieved the inhibition of downstream SNAIL by sponging miR‐370‐3p. Moreover, we found that these effects could be reversed by knocking out SNAIL. Finally, we verified that circSWT1 promoted NSCLC progression and EMT in xenograft tumor models.

**Conclusion:**

CircSWT1 promoted the invasion, migration, and EMT of NSCLC. CircSWT1 could serve as a potential biomarker and a potential therapeutic target for NSCLC.

## Graphical abstract

CircSWT1 expression was significantly upregulated in NSCLC tissues, and high expression of circSWT1 predicted poor prognosis in NSCLC via survival analysis. CircSWT1 can act as a sponge of miR‐370‐3p. Overexpression of circSWT1 induced NSCLC progression by inducing cell EMT via miR‐370‐3P/Snail axis.

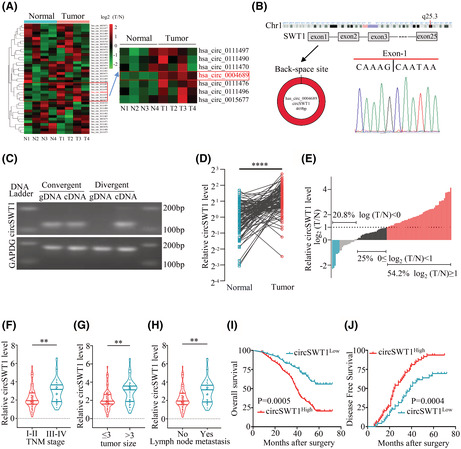

## 1 INTRODUCTION

The leading cause of cancer‐related mortality globally, with detrimental effects on human health, is lung cancer. The majority of instances of lung cancer (85%) are non‐small‐cell lung cancer (NSCLC), which is the most prevalent pathological type. With smoking cessation and improvements in early diagnosis and treatment, the death rate from lung cancer has steadily declined, and survival rates have been rapidly improving, particularly for NSCLC. All cancer patients have a 67% five‐year survival rate on average. However, patients with lung cancer have a 21% five‐year survival rate, one of the lowest among all cancer patients. Additionally, the greatest number of deaths are still from lung cancer—approximately one‐quarter of all cancer deaths are due to lung cancer.^1^ As a result, there is an urgent need for more research into the mechanism behind NSCLC progression.

Circular RNA (circRNA), a vital species of endogenous noncoding RNAs, is a form of single‐stranded RNA that mostly consists of one or more exons and has been found to be crucial in the control of transcriptional and post‐transcriptional gene activity.^2^ The transcription of precursor mRNA (pre‐mRNA) yields circRNAs through a process known as “back‐splicing”. All exons form a covalently bound closed‐loop structure without 5′–3′ polarity or a 3′ poly (A) tail, which results in a more stable character compared to linear parental genes. The biological functions of circRNAs, including miRNA sponging, interaction with proteins, translational regulation, and protein translation, have been revealed in numerous studies.^2^ For instance, circANRIL interacts with pescadillo homolog 1 (PES1) to activate p53 and produce nucleolar stress, which in turn triggers apoptosis and suppresses cell proliferation, slowing the progression of vascular atherosclerosis.^3^ Moreover, circRNAs exert a major influence on tumor metastasis and invasion by acting as miRNA “sponges”.^4^ In addition, we previously revealed that circMET controls the miR‐145‐5p/CXCL3 axis to promote NSCLC cell proliferation, metastasis, and immune evasion.^5^ Wang et al. showed that circSPARC restricts CRC cell migration and proliferation via sponging miR‐485‐3p to increase JAK2 expression and ultimately results in the accumulation of phosphorylated JAK2 (p) ‐STAT3.^6^

In the process known as epithelial‐mesenchymal transition (EMT), epithelial cells lose their unique traits and acquire those of mesenchymal cells. Growing evidence has inked EMT to many tumor functions, including tumor migration, intravasation, and resistance to therapy.^7^ Tumor cell plasticity indicates that tumor cells remain in internal states of cell development. EMT‐driven cellular plasticity is important for the progression of embryonic development, and poor development of malignant tumors occurs if cancers control EMT plasticity.^8^ There is mounting evidence that links EMT to NSCLC. For instance, in NSCLC, circPTK2 suppressed TGF‐induced EMT by targeting TIF1; this finding showed that circPTK2 might slow tumor growth by influencing EMT, offering a fresh approach to tumor treatment.^9^ According to research by Shucai Yang et al, FOXP3 stimulates EMT, tumor development, and metastasis in NSCLC by triggering the Wnt/−catenin signaling pathway.^10^ E‐cadherin is one of the markers of EMT and is known as a metastatic suppressor in tumors.^11^ SNAIL plays a crucial function in the progression of tumors as a transcriptional regulator of E‐cadherin expression.^12^ Inhibiting tumor growth and EMT through controlling Snail, circFNDC3B‐218aa, a new protein encoded by circFNDC3B, was discovered to be effective against colon cancer in prior research.^13^

CircRNAs have a significant role in the growth of many tumor types.^4^ In addition, EMT is a process that cannot be ignored during the development of tumorigenesis. The levels of thousands of circRNAs are altered in malignant cells with EMT. However, the importance of the relationship between circRNAs and EMT has not been fully elucidated. For instance, AKT exerts a positive effect on EMT, and circNRIP1 can change the degree of AKT1 expression in gastric cancer through sponging of miR‐149‐5p. In conclusion, circNRIP1 could affect cell migration and invasion through EMT.^14^ In addition, Hong et al. found that circ‐CPA4 sponges let‐7 miRNA to inhibit tumor cell mobility, invasion, and EMT in NSCLC.^15^ The above studies showed that circRNA can induce EMT in tumor cells, which in turn affects the progression of tumors.

According to earlier research, circRNAs have been linked to the progression of many different types of solid tumors. However, circSWT1 has gotten very little attention. In this study, we found that circSWT1 level was significantly upregulated among SWT1‐derived circRNAs in NSCLC, and a high level of circSWT1 predicted poor progression of NSCLC patients. Moreover, in vitro and in vivo experiments revealed that circSWT1 facilitated invasion, metastasis, and EMT of NSCLC cells. Mechanistically, we also found that circSWT1 sponged miR‐370‐3p and promoted downstream SNAIL expression to hinder tumor progression and EMT in NSCLC. However, this phenomenon was reversed when the SNAIL was knocked out. Our study confirmed that circSWT1 could promote NSCLC invasion, migration, and EMT via the miR‐370‐3p/SNAIL axis. As a result, circSWT1 may be used as a potential biomarker and therapeutic target for NSCLC.

## 2 MATERIALS AND METHODS

### 2.1 Collection of clinical samples

Ninety‐six NSCLC specimens and matched normal tissues were obtained from the Second Affiliated Hospital of Nanchang University from 2014 to 2015. After surgical excision, pathologists categorized all of the tissues, which were then all preserved at −80°C and utilized to create a tissue microarray (TMA) for immunohistochemistry (IHC) staining. Each patient gave their informed permission before surgery, and the study was given the go ahead by the Second Affiliated Hospital of Nanchang University's ethics committee.

### 2.2 Cell culture and transfection

The human normal bronchial epithelial cells (HBE) and human NSCLC cell lines (A549, NCI‐H460, PC‐9, H1703, and NCI‐H1299) were purchased from the Cell Bank of the Chinese Academy of Sciences . In DMEM and RPMI‐1640 with 10% fetal bovine serum (Gibco) and 1% penicillin–streptomycin, all the cells were grown (Yeasen). These cells were raised at 37 °C with 5% CO2 in a cell incubator (Thermo Fisher Scientific). Following the manufacturer's instructions, lentiviral vectors carrying circSWT1 and shcircSWT1 were bought from Genomeditech and transfected into A549 and H1299 cells. Genomeditech supplied the CRISPR Cas9‐gDNA technology that was utilized to eradicate the SNAIL gene from the A549 and H1299 cell lines. The sequences of shcircSWT1 are reported in Additional file 1: Table S1 and those of sgSNAIL are listed in Additional file 2: Table S2, respectively.

### 2.3 Colony formation, Matrigel transwell, wound‐healing experiments, and the cell counting kit‐8 (CCK‐8)

All these assays were described in our previous study.^16^, ^17^ Cell growth was assessed using CCK‐8 assays. ShcircSWT1 or overexpression vector‐transfected cells were incubated in 96‐well plates for 24, 48, 72, and 96 hours. OD450 values were measured.

Assays for colony formation were carried out to assess the viability of NSCLC cells. In a word, 1000 cells were added to 6‐well plates and incubated for 2 weeks. After that, we counted the colony numbers after staining.

Matrigel transwell assays were used to measure cell invasion. NSCLC cells were first suspended in serum‐free media before being put on top of chambers that had been coated with 1 μg/μl Matrigel (BD Biosciences). The bottom of the chambers was filled with a medium containing 10% FBS. The cells were then fixed with 4% paraformaldehyde after being cultured for 48 hours at 37°C and 5% CO_2_. Finally, the cells were counted after staining by 0.1% crystal violet (Sigma) via a light microscope.

Wound‐healing experiments were used to evaluate cell migration. In brief, NSCLC cells were incubated in six‐well plates until 95% confluence was obtained. Thus, we created a vertical scratch in the center of the six‐well plates and filled them with serum‐free media. Finally, migration areas were imaged and measured every 24 hours by Image J.

### 2.4 Western blotting and IHC

These experiments have been carried out in prior studies,^5^, ^16^ which are detailed in Additional file 3: Appendix S1. We show the antibody in Additional file 4: Table S3.

### 2.5 Immunofluorescence

A549‐circSWT1, H1299‐shcircSWT1 and control cells were grown on cell slides. Next, the cells were processed by fixation, permeabilization, and blocking with goat serum albumin incubation (Origene). All cells were then treated with particular primary antibodies for 12 hours at 4°C. As a result, the cells were treated for 1 hour at 37°C with a secondary antibody. DAPI (Yeasen) was added to the slides, and cells were photographed using a fluorescence confocal microscope. The antibody is presented in Additional file 4: Table S3.

### 2.6 RNA isolation and qRT‐PCR assay

RNA isolation and qRT‐PCR analysis were carried out in the same manner as in prior studies^5^, ^16^ and as detailed in Additional file 1 of the Appendix S1. We show all primers in Additional file 5: Table S4.

### 2.7 Agarose gel electrophoresis

This experiment was done to further verify the PCR products. The cDNA and gDNA reverse transcription and isolated from H1299 cells were amplified via PCR with convergent and divergent primers. Furthermore, the PCR products were run on an agarose gel. The results were analyzed by a gel imaging system (Tanon). Additional file 5: Table S4 contains the primers.

### 2.8 Dual‐luciferase reporter gene assay and fluorescence in situ hybridization (FISH)

We established pGL3‐LUC‐circSWT1, pGL3‐LUC‐SNAIL, mutant pGL3‐LUC‐circSWT1, and mutant pGL3‐LUC‐SNAIL for the dual‐luciferase reporter gene experiment. The wild‐type or mutant vectors were then co‐transfected into HEK‐293 T cells with miR‐370‐3p mimics or NC mimics using transfection reagents (Thermo Fisher, Cat: 11668–019). After 48 hours of incubation, cells were lysed and centrifuged, and cell supernatants were collected for detection. A dual‐luciferase reporter assay system was used to measure luciferase activity in cell supernatants (Promega).

For the FISH, cells were cultured on coverslips, immobilized with 4% paraformaldehyde, washed with PBS, and digested by protease K (Sangon) for 5 minutes at 37°C and 5% CO_2_. Next, cells were washed again and fixed with 1% paraformaldehyde; thus, we dehydrated these cells. The coverslips were hybridized with a specific probe at 37°C overnight. Finally, at room temperature, the coverslips were rinsed and the cell nuclei stained with DAPI. A confocal fluorescence microscope was used to take the pictures (LSM510; Zeiss). Table S5 in Additional file 6 shows all of the probes.

### 2.9 CircRNA precipitation (circRIP) and RNA immunoprecipitation (RIP)

GeneChem (Shanghai) supplied the biotin‐labeled circSWT1 probe for the circRIP assay. In short, after transfection with the biotin‐circSWT1 and NC probes, H1299 cells were fixed in 4% formaldehyde (Sigma‐Aldrich) and then subjected to a number of procedures, including lysis, sonication, and centrifugation. Thus, the supernatant was incubated for 12 hours with M280 streptavidin Dynabeads (Invitrogen). The compound was rinsed in elution buffer and suspended for 2 hours in lysis buffer. TRIzol reagent (Invitrogen) was used to get total RNA from the compound. qRT‐PCR was then used to measure the relative amount of RNA.

The Magna RIP kit was used for the RIP assay, which was carried out according to the manufacturer's instructions (Millipore). TRIzol reagent was used to extract total RNA, and qRT‐PCR was utilized to detect the levels of circSWT1, miR‐370‐3p, and circANRIL. Additional file 4: Table S3 contains information on the antibody.

### 2.10 RNA pulldown assay

The RNA pulldown experiment was carried out exactly as previously described.^16^ For 24 hours, the biotinylated miR‐370‐3p and negative control (NC) probes were transfected into A549 and H1299 cells. These cells were then lysed and mixed with M‐280 streptavidin magnetic beads (Invitrogen), which were treated for no more than 2 hours. Subsequently, the RNAs combined with the beads were extracted and the results were evaluated using qRT‐PCR.

### 2.11 In vivo assays

Male nude mice aged 5–6 weeks were acquired from Gempharmatech (Nanjing) and raised in a sterile setting in the Center for Experimental Animals of the Second Affiliated Hospital of Nanchang University. All animal tests were carried out in line with the amended Animals (Scientific Procedures) Act 1986 in the United Kingdom and Directive 2010/63/EU in Europe, and were approved by the Ethics Committee of Nanchang University's Second Affiliated Hospital. Subcutaneous injection with A549‐control, A549‐circSWT1, H1299‐control, and H1299‐shcircSWT1 was performed, and the tumors were measured every 3 days. In addition, tumors were collected from the mice after 30 days, made into paraffin sections, and stained with HE, SNAIL, E‐cadherin, N‐cadherin, and vimentin. IHC was used to screen for EMT indicators in these subcutaneous tumors.

Furthermore, A549‐circSWT1, H1299‐shcircSWT1, and control cells were injected into mouse tail veins to create pulmonary metastasis tumor models in order to analyze the metastatic capabilities of NSCLC cells affected by circSWT1.

### 2.12 Statistical analysis

SPSS 23.0 was used to analyze the data (IBM SPSS). The values are displayed as the mean ± standard deviation (SD). The significance of differences between two groups was determined using an unpaired two‐tailed Student's *t* test. To assess variations in the prognosis of patients with NSCLC, the Kaplan–Meier method and the log‐rank test were utilized, and Cox's regression model was used to study independent prognostic factors. The associations between the levels of circSWT1, miR‐370‐3p, and SNAIL were investigated using Spearman correlation analysis. All *p* values were two‐tailed, with a *p* value of 0.05 deemed statistically significant.

## 3 RESULTS

### 3.1 The relationship between the circSWT1 level and the clinicopathological characterization of NSCLC patients

**FIGURE 1:**
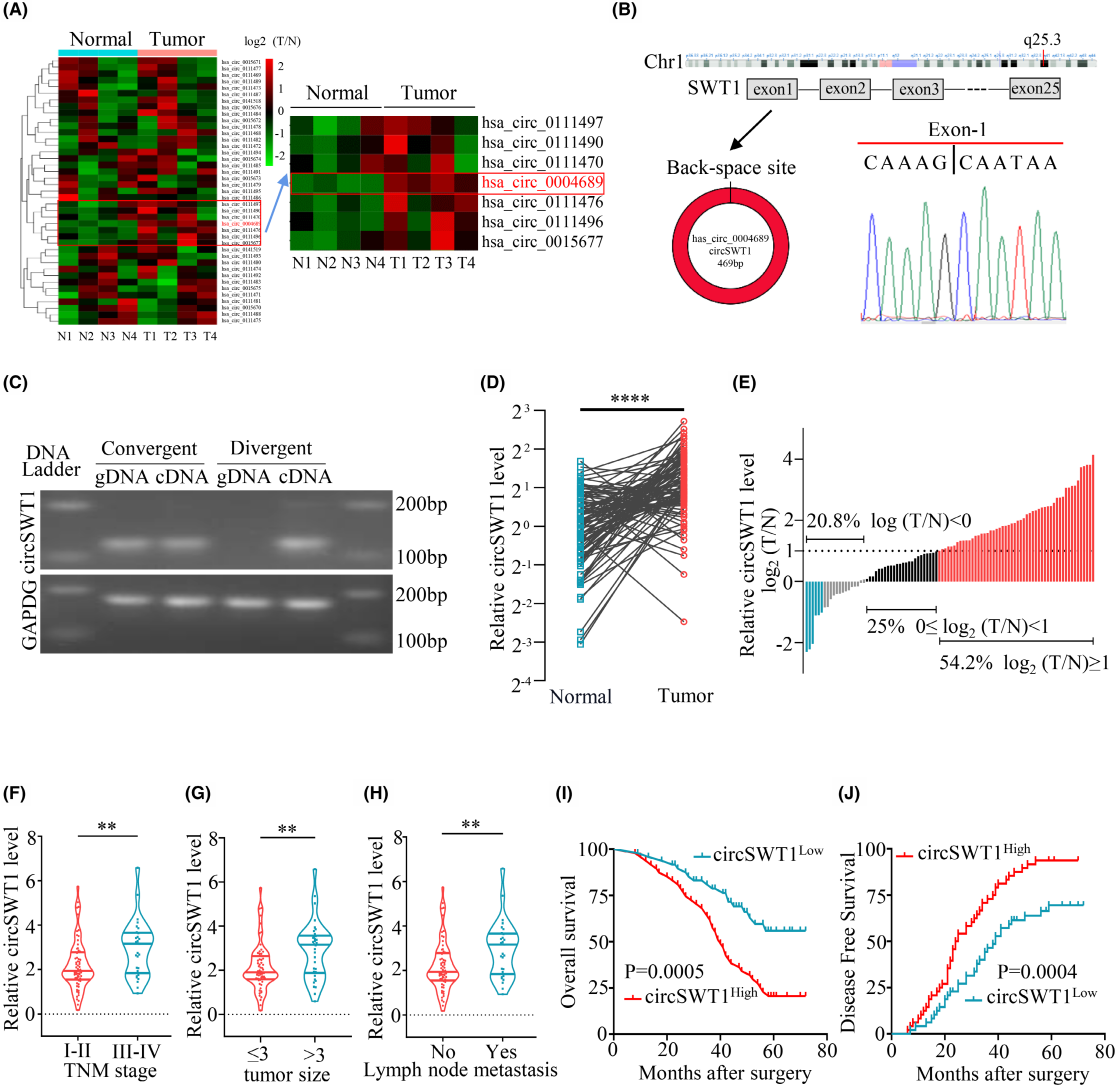
Clinicopathological characterization of circSWT1 in NSCLC patients. (A) Heatmap of SWT1‐derived circRNA levels in 4 NSCLC tissues and paired normal tissues from NSCLC patients. (B) Structural schematic diagram of circSWT1 (left) and Sanger sequencing showing the back‐splicing site of circSWT1 (right). (C) PCR results of circSWT1 with divergent or convergent primers from cDNA and gDNA. [(D) and (E)] The level of circSWT1 was measured in 96 paired NSCLC tissues (T) and paired normal tissues (N) by qRT‐PCR. (F) The relationship between circSWT1 level and TNM stage (I‐II vs. III‐IV). (G) The relationship between circSWT1 level and tumor size (≤3 cm vs. >3 cm). (H) The relationship between circSWT1 level and lymph node metastatic status (yes vs. no). [(I) and (J)] Ninety‐six tumor patients were separated into two groups based on circSWT1 level (circSWT1^High^ vs. circSWT1^Low^), and the overall survival rates and disease free survival rates of each group were calculated using Kaplan–Meier and log‐rank analyses. The data are presented as the mean ± SD of three independent experiments. **p < 0.01, ****p < 0.0001, ns: not significant.

CircRNA has been linked to the development of a number of malignancies and has been shown to alter patient prognosis, including NSCLC.^18^, ^19^, ^20^, ^21^ To investigate the functions of SWT1‐derived circRNAs in NSCLC, we used qRT‐PCR to detect the levels of circRNAs produced by SWT1 in four pairs of NSCLC and normal tissues. The findings revealed that the level of hsa_circ_0004689 (circSWT1) was dramatically increased in NSCLC tissues (Figure 1A). CircSWT1 was generated from exon 1 of SWT1, and the loop structure of circSWT1 was verified by Sanger sequencing with a divergent primer (Figure 1B). Furthermore, the results of agarose gel electrophoresis indicated that circSWT1 could only be transcribed from cDNA, not gDNA, using divergent primers (Figure 1C).

To further quantify the connection between clinicopathological features and circSWT1 in NSCLC, the level of circSWT1 was measured in 96 NSCLC and matched normal tissues using qRT‐PCR. First, the level of circSWT1 in 96 NSCLC tissues was remarkably upregulated in normal tissues (Figure 1D). Furthermore, 52 individuals had circSWT1 levels in NSCLC tissues that were twice as high as in normal tissues (Figure 1E). Table 1 shows the connections between the circSWT1 level and the clinicopathological characteristics of 96 NSCLC patients. The level of circSWT1 was substantially greater in high TNM stage patients than in low TNM stage patients (Figure 1F). In addition, according to the findings, higher circSWT1 levels were associated with larger tumor sizes and lymph node metastases in individuals with NSCLC (Figure 1G, H). Furthermore, Kaplan–Meier analysis revealed that patients with greater circSWT1 levels had worse overall survival rates and higher rates of postoperative recurrence than those with lower circSWT1 levels (Figure 1I, J). In addition, circSWT1 was found to be an independent predictor of NSCLC patient prognosis using Cox analysis (Tables 2 and 3). In a word, the level of circSWT1 in NSCLC tissues was dramatically elevated, and a high level of circSWT1 was related with a bad prognosis of NSCLC.

**TABLE 1:** Correlations between circRNA SWT1 and clinicopathological features in 96 NSCLC patients

Variables	CircSWT1 expression level		*p* value
	Low	High	
Age			
<60	26	27	0.837
≥60	22	21	
Gender			
Male	29	26	0.536
Female	19	22	
Smoking history			
Smokers	21	26	0.307
Nonsmokers	27	22	
Tumor diameter			
≤3	36	26	0.033
>3	12	22	
Tumor stage			
I–II	38	29	0.045
III–IV	10	19	
Lymph node metastasis			
Yes	9	20	0.014
No	39	28	
Differentiation			
Well and moderate	18	25	0.151
Poor	30	23	

**TABLE 2:** Univariate and multivariate analyses of factors associated with overall survival

Factors	OS					
	Univariate			Multivariate		
	HR	95% CI	*p* Value	HR	95% CI	*p* Value
Age(<60 vs. ≥60)	1.578	0.927–2.685	0.093			NA
Gender(male vs. female)	0.730	0.430–1.240	0.245			NA
Smoking history (smokers vs. nonsmokers)	0.798	0.469–1.358	0.406			NA
Tumor diameter (≤3 cm vs. >3 cm)	0.444	0.259–0.761	0.003			NS
Tumor stage (I–II vs. III–IV)	1.889	1.096–3.258	0.022			NS
Lymph node metastasis (yes vs. no)	1.869	1.082–3.230	0.025			NS
Differentiation (well and moderate vs. poor)	0.727	0.428–1.236	0.239			NA
CircSWT1 expression (High vs. Low)	2.600	1.478–4.572	0.001	2.104	1.154–3.836	0.015

**TABLE 3:** Univariate and multivariate analyses of factors associated with disease free survival

Factors	DFS					
	Univariate			Multivariate		
	HR	95% CI	*p* value	HR	95% CI	*p* value
Age(<60 vs. ≥60)	1.207	0.769–1.893	0.413			NA
Gender(male vs. female)	0.835	0.531–1.313	0.436			NA
Smoking history (smokers vs. nonsmokers)	0.883	0.564–1.381	0.585			NA
Tumor diameter (≤3 cm vs. >3 cm)	2.182	1.361–3.498	0.001			NS
Tumor stage (I–II vs. III–IV)	1.708	1.062–2.749	0.027			NS
Lymph node metastasis (Yes vs. No)	1.974	1.227–3.174	0.005			NS
Differentiation (well and moderate vs. poor)	0.868	0.554–1.361	0.537			NA
CircSWT1 expression (high vs. low)	2.214	1.397–3.507	0.001	1.776	1.081–2.917	0.023

### 3.2 The overexpression of circSWT1 promotes the viability, invasion, and migration of NSCLC cells

**FIGURE 2:**
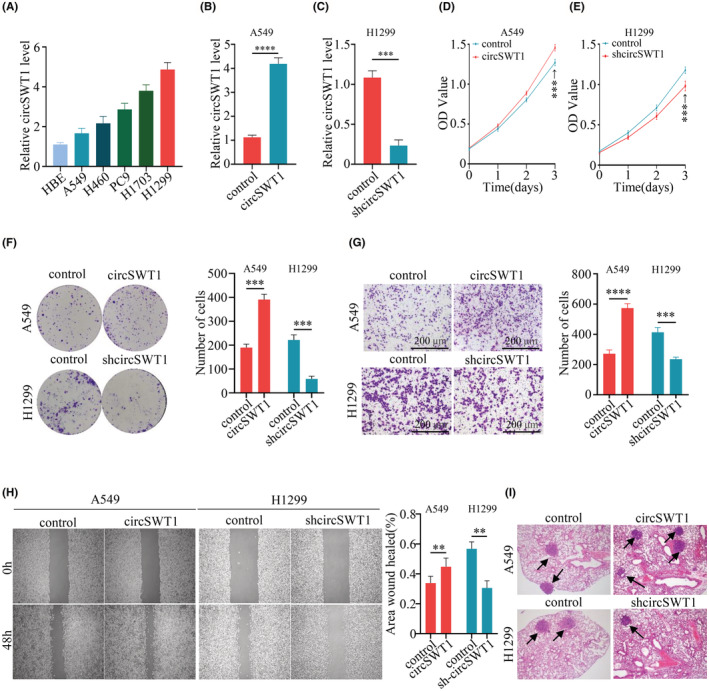
Overexpression of circSWT1 promotes the viability, invasion, and migration of NSCLC cells. (A) The level of circSTW1 was lowest in A549 cells and highest in H1299 cells. [(B) and (C) The transfection efficiency of two stably transfected cell lines (A549‐circSWT1 and H1299‐shcircSWT1) was measured by qRT‐PCR. [(D) and (E)]. The viability of A549‐circSWT1 and H1299‐shcircSWT1 cells was assessed by CCK‐8 assays. (F) The viability of A549‐circSWT1 and H1299‐shcircSWT1 cells was assessed by colony formation assays. (G) The invasion of A549‐circSWT1 and H1299‐shcircSWT1 cells was evaluated using Matrigel Transwell assays. (H) The migration of A549‐circSWT1 and H1299‐shcircSWT1 cells was assessed by wound healing assays. (I) The number of lung metastases was higher in the A549‐circSWT1 group and lower in the H1299‐shcircSWT1 group than in the control group. The data are presented as the mean ± SD of three independent experiments. **p < 0.01, ***p < 0.001, ****p < 0.0001, ns: not significant.

Given the probable relevance of circSWT1 in NSCLC patient prognosis, we conducted a series of studies to evaluate circSWT1's biological roles in NSCLC. qRT‐PCR was used to measure the levels of circSWT1 in HBE cells and 5 NSCLC cells (A549, NCI‐H460, PC‐9, H1703, and NCI‐H1299). The results showed that circSWT1 levels were highest in H1299 cells and lowest in A549 cells (Figure 2A). As a result, we developed two stably transfected cell lines: A549‐circSWT1 and H1299‐shcircSWT1. qRT‐PCR was used to assess transfection efficiency (Figure 2B, C).

Moreover, further studies were conducted to verify whether circSWT1 affects the development of tumors. First, we found that overexpression of circSWT1 could promote the viability of A549 cells and knockdown of circSWT1 inhibited tumor viability, according to CCK‐8 and colony formation assay results (Figure 2D–F). Matrigel Transwell assay findings revealed that A549‐circSWT1 cell invasion was increased, whereas H1299‐shcircSWT1 cell invasion was inhibited (Figure 2G). Migration of A549‐circSWT1 cells was greatly enhanced; migration of H1299‐shcircSWT1 cells was dramatically inhibited, as confirmed by wound healing assays (Figure 2H). Finally, we constructed mouse models of lung metastases by tail vein injection. We found more pulmonary metastatic tumors in the mice injected with A549‐circSWT1 cells than in the mice injected with H1299‐shcircSWT1 cells (Figure 2I).

### 3.3 The overexpression of circSWT1 promotes EMT in NSCLC

**FIGURE 3:**
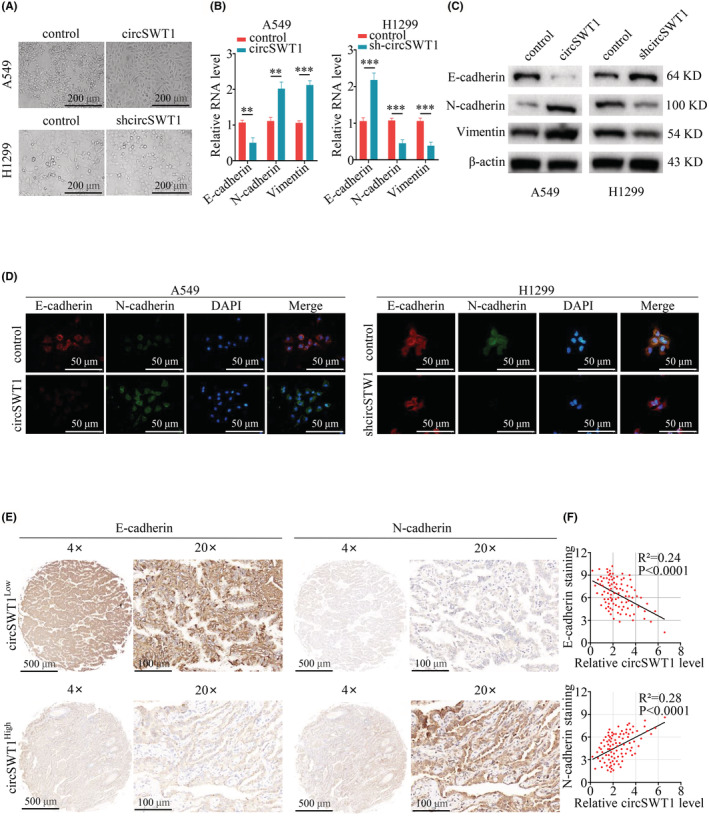
The overexpression of circSWT1 promotes EMT in NSCLC. (A) A549 cells overexpressing circSWT1 displayed a needle‐like morphology, but H1299 cells with circSWT1 knockdown presented no significant differences. (B) Relative RNA level of E‐cadherin, N‐cadherin, and vimentin in A549 (A549‐control and A549‐circSWT1) and H1299 (H1299‐control and H1299‐shcircSWT1) cells was measured by qRT‐PCR. (C) Relative protein levels of E‐cadherin, N‐cadherin and imentin in A549 (A549‐control and A549‐circSWT1) and H1299 (H1299‐control and H1299‐shcircSWT1) cells were assessed by western blotting. (D) Immunofluorescence staining of E‐cadherin and N‐cadherin in A549 (A549‐control and A549‐circSWT1) and H1299 (H1299‐control and H1299‐shcircSWT1) cells. (E) The paraffin sections were split into two groups based on circSWT1 level, and IHC was performed to assess the levels of E‐cadherin and N‐cadherin in the two groups. (F) The correlation between circSWT1 and E‐cadherin and N‐cadherin was analyzed. CircSWT1 level was measured by qRT‐PCR, and the level of E‐cadherin and N‐cadherin was measured by IHC. The data are presented as the mean ± SD of three independent experiments. **p < 0.01, ***p < 0.001.

The above results showed that circSWT1 is significantly associated with NSCLC, and a previous study reported that EMT is a critical link to the development of NSCLC,^9^ so the relationship between circSWT1 and cell EMT needs further study. The cell morphology changed from normal to needle‐like in A549‐circSWT1 cells, as shown by a light microscope (100×). However, there was no obvious difference between H1299‐shcircSWT1 and H1299‐control cells (Figure 3A). Next, the relative RNA level of EMT markers was measured by qRT‐PCR. E‐cadherin levels were lower in the A549‐circSWT1 group and greater in the H1299‐shcircSWT1 group compared to the control group, whereas N‐cadherin and Vimentin levels exhibited the reverse tendency (Figure 3B). Then, using Western blotting, we examined EMT‐related proteins such as E‐cadherin, N‐cadherin, and vimentin. The results showed that the E‐cadherin level was significantly lower and the N‐cadherin and vimentin levels were significantly higher in the A549‐circSWT1 group compared to the A549‐control group. In the H1299 group, however, the reverse tendency was found (H1299‐control vs. H1299‐shcircSWT1) (Figure 3C). Moreover, E‐cadherin and N‐cadherin FISH staining in A549‐circSWT1 cells revealed that E‐cadherin was low and N‐cadherin was high. Instead, FISH staining of H1299‐shcircSWT1 cells revealed that E‐cadherin levels were high while N‐cadherin levels were low (Figure 3D). In addition, we split NSCLC patients into two groups depending on their circSWT1 levels. IHC was used to assess the levels of E‐cadherin and N‐cadherin. The results indicated that the circSWT1^High^ group had higher levels of E‐cadherin and lower levels of N‐cadherin than the circSWT1^Low^ group. CircSWT1 levels were shown to be positively correlated with E‐cadherin levels and negatively correlated with N‐cadherin levels (Figure 3E, F).

### 3.4 CircSWT1 upregulates the expression of downstream SNAIL by sponging miR‐370‐3p

**FIGURE 4:**
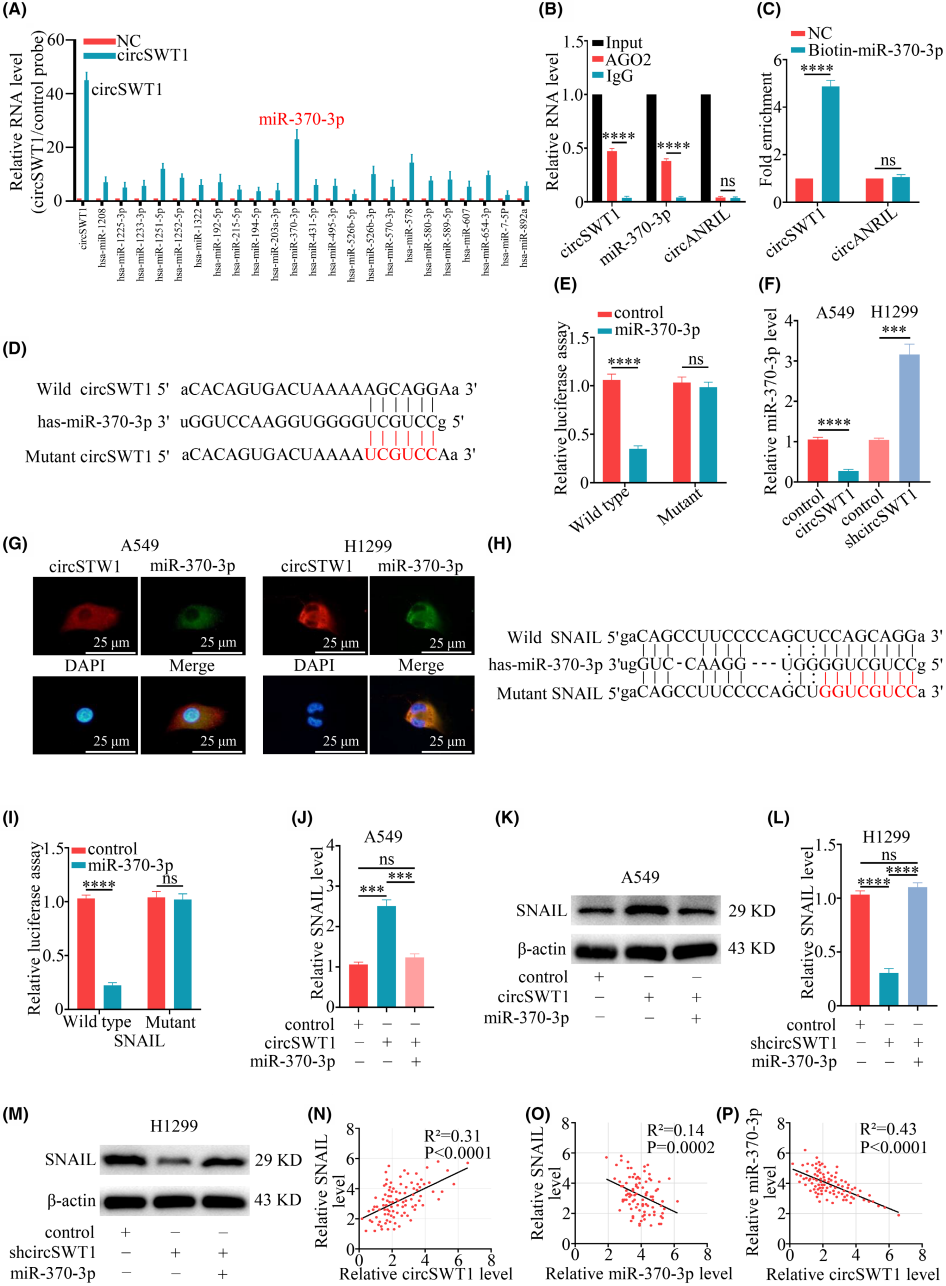
CircSWT1 upregulates the expression of the downstream molecule SNAIL by sponging miR‐370‐3p. (A) The relative RNA level of H1299 cells was measured with a biotinylated circSWT1 probe in the circRIP assay. (B) The RIP assay was used to verify the interaction between circSWT1 and miR‐370‐3p by using the anti‐AGO2 antibody in H1299 cells. (C) The enrichment of circSWT1 in H1299 cells transfected with biotinylated miR‐370‐3p mimics was measured by the RNA pulldown assay, and circANRIL acted as a negative control. (D) The potential binding sites between circSWT1 and miR‐370‐3p. (E) The luciferase activity of wild‐type and mutant circSWT1 in HEK‐293 T cells transfected with miR‐370‐3p mimics. (F) The level of miR‐370‐3p significantly decreased in A549 cells with circSWT1 overexpression and significantly increased in H1299 cells with circSWT1 knockdown compared to the controls. (G) The images of colocalization between circSWT1 and miR‐370‐3p in A549 and H1299 cells via RNA FISH staining were captured with a fluorescence microscope. Nuclei were stained with DAPI. (H) The putative binding sites between SNAIL and miR‐370‐3p. (I) The luciferase activity of SNAIL (wild‐type and mutant type) was measured in HEK‐293 T cells transfected with miR‐370‐3p mimics. (J) The expression of SNAIL was quantified with qRT‐PCR after the dual overexpression of circSWT1 and miR‐370‐3p in A549 cells. (K) The expression of SNAIL was estimated with Western blotting after the dual overexpression of circSWT1 and miR‐370‐3p in A549 cells. (L) The expression of SNAIL was quantified with qRT‐PCR after the dual knockdown of circSWT1 and miR‐370‐3p in H1299 cells. (M) The expression of SNAIL was estimated with Western blotting after the dual knockdown of circSWT1 and miR‐370‐3p in H1299 cells. [(N)–(P)] The correlations among the levels of circSWT1, miR‐370‐3p and SNAIL in 96 NSCLC patients were assessed by qRT–PCR. The data are presented as the mean ± SD of three independent experiments. ***p < 0.001, ****p < 0.0001, ns: not significant.

Numerous studies have revealed that circRNAs play an important role in sponging miRNAs in malignancies, including NSCLC.^4^ We anticipated the miRNAs that may be targeted by circSWT1 using the circular RNA interactome website. Then, we used the biotinylated circSWT1 probe to search for relevant miRNAs in H1299 cells via circRIP, and qRT‐PCR revealed an obvious enrichment of miR‐370‐3p (Figure 4A). RIP with an anti‐AGO2 antibody was used to examine the significant enrichment of circSWT1 and miR‐370‐3p, revealing the interaction between circSWT1 and miR‐370‐3p (Figure 4B). CircSWT1 was significantly enriched in the biotinylated miR‐370‐3p RNA pulldown assay (Figure 4C). Then, the wild‐type (pGL3‐LUC‐circSWT1) and mutant vectors (pGL3‐LUC‐circSWT1) were co‐transfected into HEK‐293 T cells with miR‐370‐3p mimics or NC mimics. According to the dual‐luciferase reporter experiment, miR‐370‐3p mimics significantly decreased luciferase activity in the circSWT1‐WT group (Figure 4D, E). Furthermore, when compared to control cells, the level of miR‐370‐3p was significantly lower in A549‐circSWT1 cells and markedly higher in H1299‐shcircSWT1 cells (Figure 4F). CircSWT1 and miR‐370‐3p were found colocalized in the cytoplasm of A549 and H1299 cells using the RNA FISH technique (Figure 4G).

Then, we elucidated whether miR‐370‐3p affected NSCLC cell phenotype with or without circSWT1. A549‐circSWT1‐control mimics cells displayed considerably different cell morphologies than A549‐circSWT1‐miR‐370‐3p mimics cells under a light microscope (100). (Figure S1A). According to CCK‐8 assay results, we discovered that circSWT1 overexpression may increase A549 cells viability, and that miR‐370‐3p could counteract this impact on tumor viability (Figure S1B). In addition, wound healing and Matrigel Transwell assays demonstrated that overexpression of circSWT1 promoted the migration and invasion of A549 cells, but miR‐370‐3p counteracted this effect (Figure S1C, D).

Next, the possible binding targets of miR‐370‐3p were then predicted using StarBase 3.0, miRmap, and PITA. The results showed that SNAIL contained target sequences for miR‐370‐3p (Figure 4H) and was inextricably linked to the evolution of EMT. Following that, pGL3‐SNAIL‐WT and pGL3‐SNAIL‐Mut were co‐transfected with either a miR‐370‐3p mimic or an NC mimic, and the luciferase reporter experiment revealed that the wild‐type SNAIL sequence had much greater luciferase activity than the mutant SNAIL sequence (Figure 4I). The expression of SNAIL was dramatically increased after overexpression of circSWT1 and restored after further overexpression of miR‐370‐3p, as shown by western blotting and qRT‐PCR of A549 cells (Figure 4J, K). Similarly, in H1299 cells, the expression of SNAIL was significantly reduced after knocking down circSWT1, but this effect was eliminated after further knockdown of miR‐370‐3p (Figure 4L, M). Additionally, in 96 NSCLC patients, we assessed the levels of circSWT1, miR‐370‐3p, and SNAIL to analyze the interactions between them. According to the results, miR‐370‐3p level was negatively associated with circSWT1 and SNAIL, whereas circSWT1 level was positively associated with SNAIL (Figure 4N–P). Consequently, by sponging miR‐370‐3p, circSWT1 eliminates the inhibition of downstream SNAIL and then upregulates SNAIL expression in NSCLC.

### 3.5 The effect of circSWT1 on tumor progression and EMT is reversed by knocking out SNAIL

**FIGURE 5:**
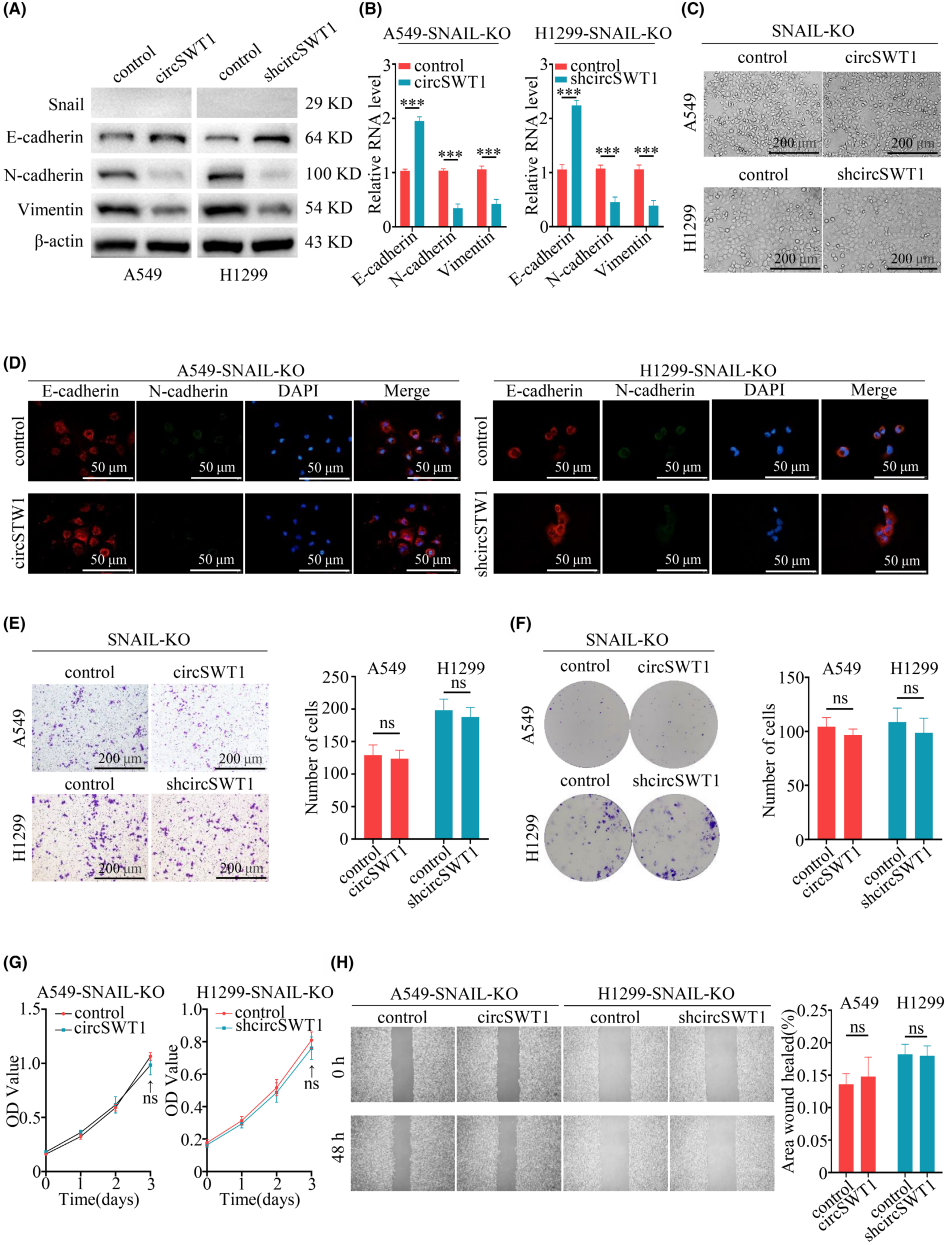
The effect of circSWT1 on the progression of tumors and EMT is reversed by knocking out SNAIL in NSCLC. (A) Relative protein levels of E‐cadherin, N‐cadherin, and vimentin were assessed by Western blotting of A549 (A549‐control and A549‐circSWT1) and H1299 (H1299‐control and H1299‐shcircSWT1) cells with SNAIL knockout. (B) qRT‐PCR was used to determine the relative RNA levels of E‐cadherin, N‐cadherin, and vimentin in A549 (A549‐control and A549‐circSWT1) and H1299 (H1299‐control and H1299‐shcircSWT1) cells with SNAIL knockout. (C) The deletion of SNAIL in A549‐circSWT1 and H1299‐shcircSWT1 cells did not result in a spindle‐like shape. (D) Immunofluorescence staining of E‐cadherin and N‐cadherin in A549 (A549‐control and A549‐circSWT1) and H1299 (H1299‐control and H1299‐shcircSWT1) cells with SNAIL knockout. (E) Matrigel Transwell assays were performed to assess the invasion of A549‐circSWT1 and H1299‐shcircSWT1 cells with SNAIL knockout. (F) Colony formation assays and (G) CCK‐8 assays were performed to evaluate the viability of A549‐circSWT1 and H1299‐shcircSWT1 cells with SNAIL knockout. (H) The migration of A549‐circSWT1 and H1299‐shcircSWT1 cells with SNAIL knockout was not significantly different from that of the control group. The data are presented as the mean ± SD of three independent experiments. ***p < 0.001, ns: not significant.

The above results indicated that circSWT1 affects tumor progression and EMT and that circSWT1 can increase SNAIL expression by sponging miR‐370‐3p. SNAIL has been linked to tumor development and EMT in previous researches.^22^, ^23^ Therefore, we hypothesized that inactivating SNAIL would reverse EMT and decrease NSCLC viability, invasion, and migration. Thus, we used the CRISPR‐Cas9 method to completely knock out SNAIL in A549 (A549‐control and A549‐circSWT1) and H1299 (H1299‐control and H1299‐shcircSWT1) cells. In A549‐circSWT1 and H1299‐shcircSWT1 cells with SNAIL deletion, N‐cadherin and vimentin levels were much lower than in the control group, whereas E‐cadherin levels were significantly higher (Figure 5A). qRT‐PCR demonstrated that E‐cadherin levels were elevated while N‐cadherin and vimentin levels were lowered in both A549‐circSWT1‐SNAIL‐KO and H1299‐shcircSWT1‐SNAIL‐KO cells (Figure 5B). Next, we observed the cell morphologies of the A549‐circSWT1‐SNAIL‐KO and H1299‐shcircSWT1‐SNAIL‐KO cells using a light microscope (100×), which revealed no significant difference (Figure 5C). Moreover, immunofluorescence of E‐cadherin and N‐cadherin in the A549‐circSWT1‐SNAIL‐KO and H1299‐shcircSWT1‐SNAIL‐KO groups showed that there was no significant disparity (Figure 5D).

Furthermore, we conducted several experiments to evaluate the effects of circSWT1 in the development of NSCLC tumors. The findings revealed that circSWT1 had no influence on the viability, invasion, or migration of A549‐circSWT1‐SNAIL‐KO and H1299‐shcircSWT1‐SNAIL‐KO cells (Figure 5E–H). Overall, we found that circSWT1 influenced the EMT and progression of NSCLC by relying on SNAIL.

### 3.6 Highly expressed circSWT1 promotes the tumor progression and EMT of NSCLC in mouse models

**FIGURE 6:**
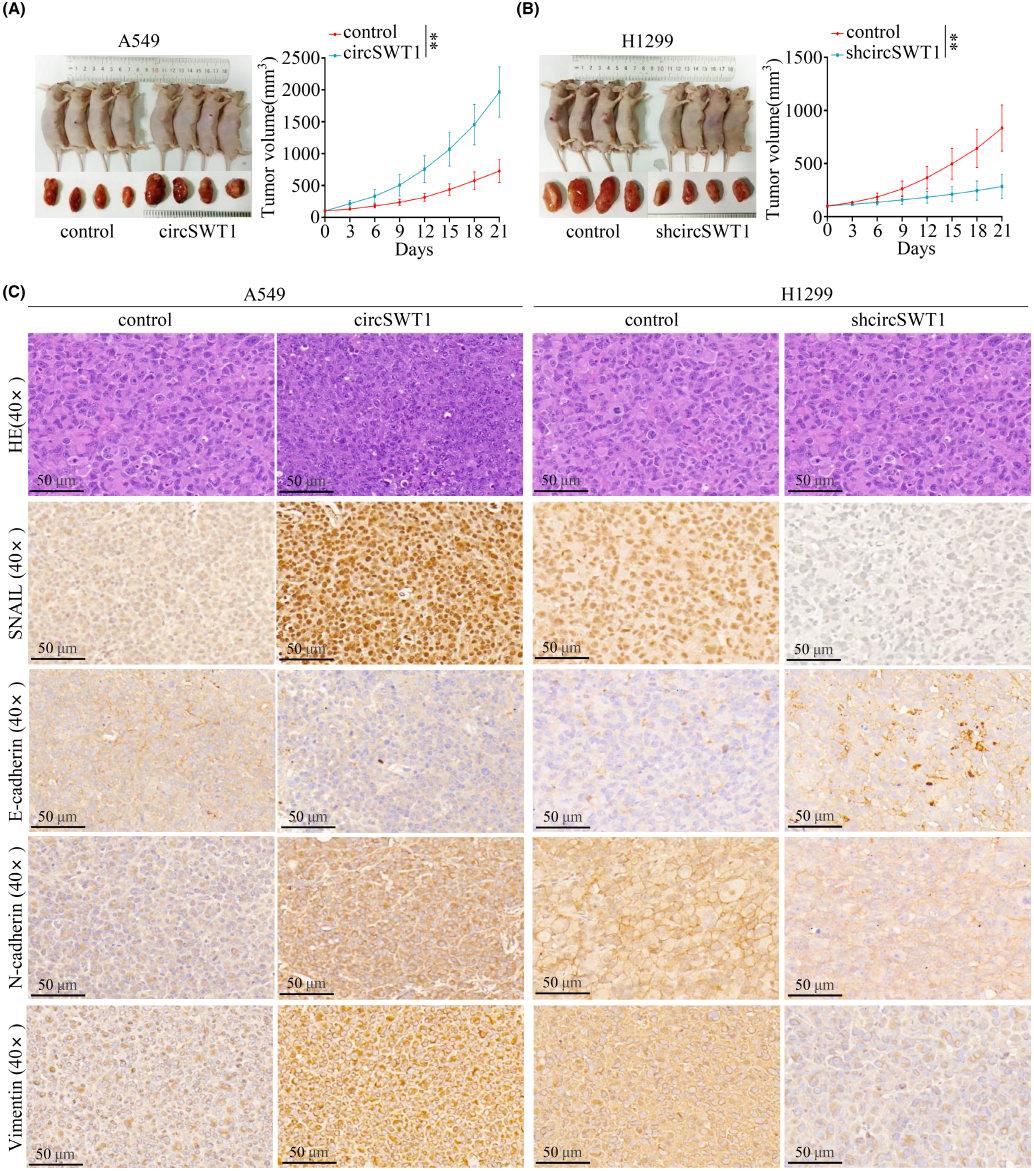
High circSWT1 level promotes NSCLC tumor progression and EMT in nude mouse models. (A) Nude mice were subcutaneously injected with A549 (A549‐control and A549‐circSWT1) cells and (B) H1299 (H1299‐control and H1299‐shcircSWT1) cells. The tumor size was measured every 3 days. (C) HE staining and IHC staining of SNAIL, vimentin, E‐cadherin, and N‐cadherin in subcutaneous tumors derived from mice of the A549 group (A519‐control and A549‐circSWT1) and H1299 group (H1299‐control and H1299‐shcircSWT1). The data are presented as the mean ± SD of three independent experiments. **p < 0.01.

Using in vitro experiments, we looked at the involvement of circSWT1 in NSCLC tumor growth and EMT, and then we constructed mouse models to confirm the role of circSWT1 in vivo. First, subcutaneous injections of A549 (A549‐control, A549‐circSWT1) and H1299 (H1299‐control, H1299‐shcircSWT1) cells were given to nude mice. The results demonstrated that overexpression of circSWT1 in A549 cells promoted tumor growth. In contrast, knockdown of circSWT1 in H1299 cells inhibited tumor growth (Figure 6A, B). Furthermore, subcutaneous tumors collected from mice were made into paraffin sections. IHC revealed that the expression of SNAIL and E‐cadherin was enhanced in the A549‐circSWT1 group, whereas N‐cadherin and vimentin were reduced in the A549‐control group. The H1299‐shcircSWT1 group and the H1299‐control group showed opposing patterns (Figure 6C).

## 4 DISCUSSION

CircRNAs were discovered nearly 50 years ago,^24^ and they were actively researched when Stanford University academics discovered a huge number of circRNAs in diverse cells using high‐throughput sequencing techniques in 2012.^25^ According to previous studies, disorders of circRNA level are associated with many human conditions, including viral infections,^26^ cardiac fibrosis,^27^ hyperglycemia^28^ and tumors.^29^ Since differences in circRNA levels were first discovered in colorectal cancer cells, the important functions of circRNAs related to the progression of tumors and EMT have been constantly explored.^30^, ^31^ However, more research is needed to understand the underlying molecular mechanisms.^29^, ^32^

Previous research has shown that circRNAs play a significant role in the progression of solid tumors, including NSCLC. Moreover, little is currently known about circSWT1. As a result, in four pairings of NSCLC tumor and normal tissues, we detected circRNAs that were derived from SWT1. Tumor tissues usually have high levels of circSWT1, while paired normal tissues have lower levels. Importantly, high levels of circSWT1 decreased overall survival rates and postoperative recurrence rates in NSCLC patients. In addition, we discovered that circSWT1 not only affects tumor, invasion, and metastasis but also induces EMT by acting on the miR‐370‐3p/SNAIL axis in NSCLC. Moreover, the nude mouse subcutaneous tumor model confirmed that circSWT1 can promote tumor growth. We concluded, based on the aforementioned findings, that circSWT1 could promote tumor progression and EMT via the miR‐370‐3p/SNAIL axis in NSCLC.

Numerous researches had been conducted to investigate the biological activities of circRNAs.^2^, ^17^ Thus far, we have only found a minor fraction of the functions of circRNAs, and they have mostly been proposed to act as sponges for miRNA.^33^ MiRNAs contain nucleotide sequences that can bind to target gene mRNAs, which can inhibit the degradation of mRNAs and affect gene expression.^34^ According to several studies, circRNAs function as competitive endogenous RNAs (ceRNAs), which can bind to miRNAs via microRNA response elements (MREs) to decrease the expression of downstream target genes in malignancies.^35^ For example, circACVR2A inhibits tumor progression by interacting with miR‐626 and promotes target EYA4 expression in bladder cancer.^36^ CircRNA‐5692, according to Zhenguo Liu et al,^37^ slows the development of hepatocellular carcinoma by sponging miR‐328‐5p to promote downstream DAB2IP expression. In our study, circSWT1 promoted tumor development and EMT in NSCLC by acting as a miR‐370‐3p sponge to promote SNAIL expression. Through the website Circular RNA Interactome, we predicted that circSWT1 interacts with miR‐370‐3p to contribute to the development of malignancies, and this hypothesis was confirmed experimentally. Moreover, we predicted that SNAIL is the potential miR‐370‐3p target gene and its binding sites via StarBase 3.0, miRmap, and PITA. We thus hypothesized that circSWT1 is a possible prognostic marker for NSCLC and that it exerts its tumor suppressive impact via the miR‐370‐3p/SNAIL axis.

EMT is closely related to tumor metastasis, and Snail is a crucial EMT inducer. EMT is initially triggered in tumor cells, which then separate from the tumor and enter the circulation to promote metastasis. This process has been seen in several human malignancies, including breast,^38^, ^39^ head and neck,^40^ gastric,^41^ and lung.^42^ The Snail/Slug family, Twist, EF1/ZEB1, SIP1/ZEB2, and E12/E47 are a few transcription factors that function as molecular switches in the EMT program.^43^, ^44^, ^45^ EMT is tightly correlated with the levels of E‐cadherin, N‐cadherin, and vimentin. When tumor cells undergo EMT, E‐cadherin is replaced by N‐cadherin that can provide greater ligation flexibility. Vimentin is a crucial component of the cytoskeleton, and the level of vimentin diminishes when the cell shape changes during EMT. This change results in cell isolation and increased cell motility. Snail, a vital transcriptional repressor of E‐cadherin, includes three family proteins: Snail1 (Snail), Snail2 (Slug), and Snail3 (Smuc). Both an N‐terminal domain and a C‐terminal domain are present in these Snail family members. The C‐terminal domain has four to six zinc fingers and binds to the E‐box motif (5′‐CANNTG‐3′) in target gene promoters. The N‐terminal domain contains the SANG (Snail/Gfi) domain, which is necessary for Snail to interact with various signaling molecules and start the EMT process.^12^ Snail has been identified as a key regulator of the EMT signaling pathway and its tight association with tumor metastasis has been confirmed. For example, Snail was shown to be required for metastatic lesions in human breast carcinoma and ovarian cancer.^23^, ^46^ Additionally, Snail has been confirmed to participate in the invasion and recurrence of tumors.^47^, ^48^, ^49^ In the present research, we discovered that knocking out SNAIL could inhibit tumor progression and EMT in vitro.

Snail is a critical target for pharmaceutical agents' development. Given the significant roles of Snail in many types of tumors, Snail inhibitors may be candidates to inhibit tumor progression and recurrence. According to earlier studies, Co (III)‐Ebox is a potent inhibitor of Snail‐mediated transcriptional suppression in breast cancer cells. Zinc finger domain‐containing Ebox‐binding proteins like Snail are inhibited by Co (III)‐Ebox, which reduces Snail activity without changing Snail protein levels. These findings revealed that this Co (III)‐DNA conjugate has beneficial therapeutic effects as an inhibitor of Snail‐induced tumor development and recurrence.^49^ In addition, a small‐molecule substance called CYD19 was found by Hong‐Mei Li et al. to bind to Snail and prevent it from interacting with CREB‐binding protein (CBP)/p300. This action impairs Snail acetylation by CBP/p300 and eventually leads to Snail protein breakdown. Furthermore, these researchers found that CYD19 plays a significant part in reversing Snail‐mediated EMT and may diminish the damage of EMT‐associated invasion and metastasis. Drugs that target Snail through CYD19 may therefore have a potential therapeutic role in cancer patients.^50^

In this study, we discovered that the level of circSWT1 was highly elevated in NSCLC and that a high level of circSWT1 was linked to a poor prognosis in NSCLC patients. Through the use of multivariate Cox analysis, circSWT1 was discovered to be a standalone predictor of the prognosis of NSCLC patients. According to these results, circSWT1 could serve as a prognostic biomarker for NSCLC. Additionally, circSWT1 overexpression facilitated NSCLC invasion, migration, and EMT both in vivo and in vitro. Moreover, knockdown of circSWT1 prevented the invasion, migration, and EMT processes of NSCLC in vivo and in vitro. Mechanistically, circSWT1 relieved the inhibition of downstream SNAIL by sponging miR‐370‐3p. Moreover, the biological function of circSWT1 relies on downstream SNAIL, which was verified by knocking out SNAIL in A549 and H1299 cells. SNAIL has been widely studied as a therapeutic target, and inhibitors of SNAIL such as Co (III)‐Ebox and CYD19 have been widely used. Thus, circSWT1 may also be a potential therapeutic target for NSCLC.

## 5 CONCLUSION

As a result of this work, circSWT1 was identified as a possible prognostic biomarker for NSCLC and was shown to have a crucial role in the progression and EMT of NSCLC. circSWT1 may also represent a fresh therapeutic target for NSCLC.

## AUTHOR CONTRIBUTIONS

**Xiang Long:** Conceptualization (supporting); data curation (lead); investigation (equal); methodology (equal); resources (supporting); supervision (supporting); validation (lead); writing – original draft (equal). **Ding‐Guo Wang:** Data curation (supporting); formal analysis (equal); investigation (equal); software (equal); visualization (lead); writing – original draft (equal). **Zhi‐Bo Wu:** Data curation (supporting); formal analysis (supporting); software (equal); validation (supporting); visualization (supporting). **Zhong‐Min Liao:** Investigation (supporting); software (equal); visualization (supporting). **Jian‐Jun Xu:** Conceptualization (lead); funding acquisition (lead); methodology (equal); project administration (lead); resources (lead); supervision (lead); writing – review and editing (lead).

## FUNDING INFORMATION

The following grants helped to fund this research: the National Natural Science Foundation of China (82060064).

## CONFLICT OF INTEREST

The authors declare that they have no competing interests.

## ETHICAL APPROVAL

The Second Affiliated Hospital of Nanchang University Research Ethics Committee granted ethical permission (NO.SYXK2015‐0001), and each patient provided written informed consent.

## CONSENT FOR PUBLICATION

Not applicable.

## Supporting information


Figure S1.

Tables S1–S5.

Appendix S1.

## Data Availability

This published article and its additional information files contain all data produced or analyzed during this investigation.
