# Supplementary figures and images for: Circular RNA hsa_circ_0004689 (circSWT1) promotes NSCLC progression via the miR‐370‐3p/SNAIL axis by inducing cell epithelial‐mesenchymal transition (EMT)
Source: Cancer Med. 2022 Dec 19;12(7):8289–305. doi: 10.1002/cam4.5527 (PMC10134258; doi:10.1002/cam4.5527)

Figure S1

A

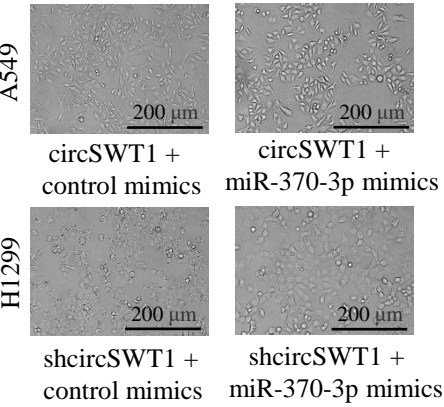

B

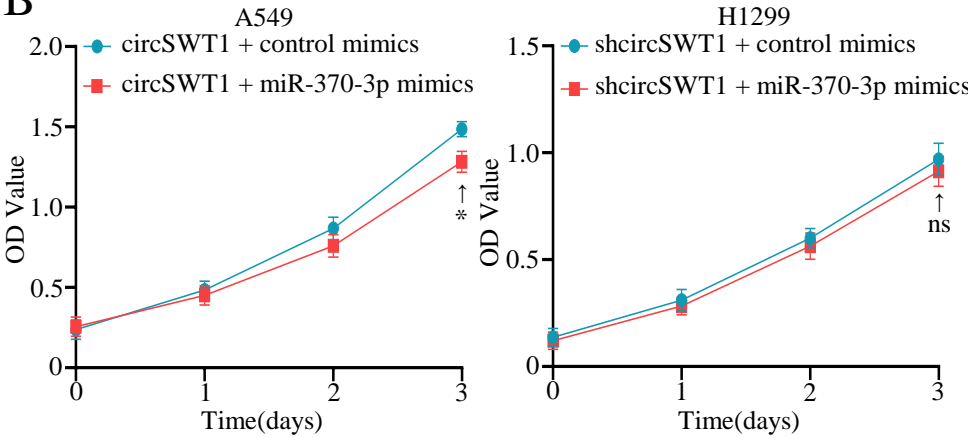

C

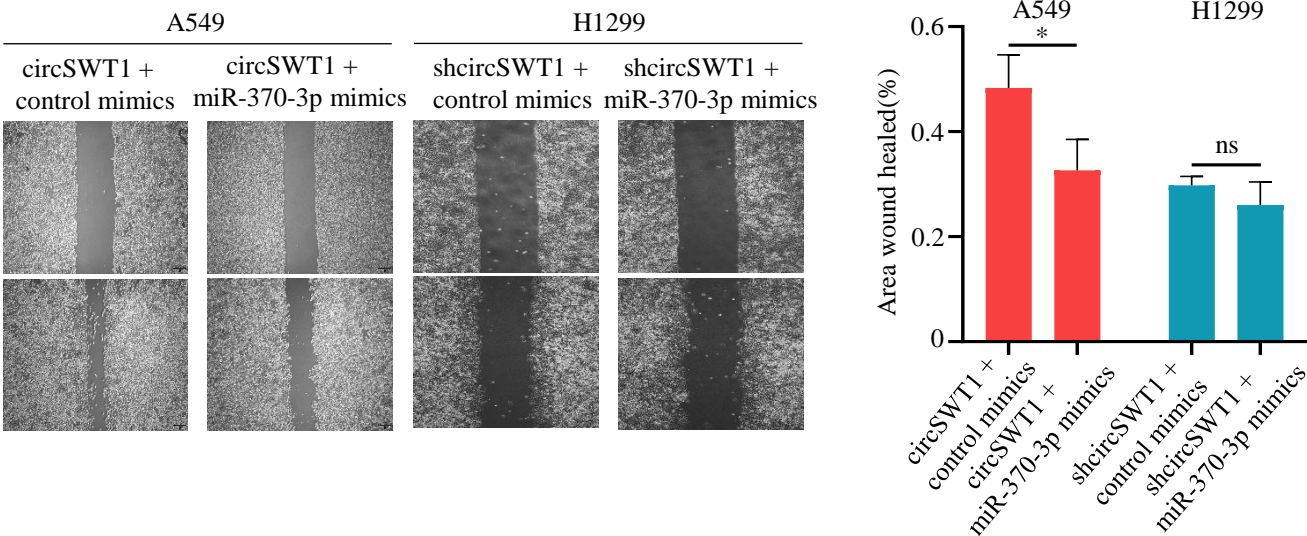

D

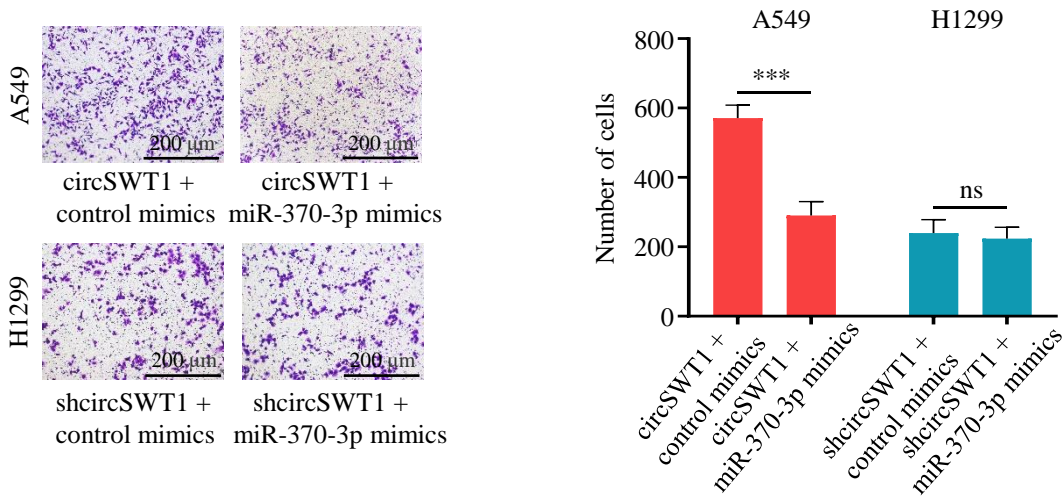

Supplement: Supplementary file 1 — Figure S1. [file CAM4-12-8289-s003.pdf]
