# Supplementary material for: Circular RNA hsa_circ_0004689 (circSWT1) promotes NSCLC progression via the miR‐370‐3p/SNAIL axis by inducing cell epithelial‐mesenchymal transition (EMT)
Source: Cancer Med. 2022 Dec 19;12(7):8289–305. doi: 10.1002/cam4.5527 (PMC10134258; doi:10.1002/cam4.5527)
Supplement: Supplementary file 2 — Tables S1–S5. [file CAM4-12-8289-s001.docx]

Supplementary table 1 The sequence of shcircSWT1

| **shRNA** | **Target sequence** |
| --- | --- |
| shRNA-1 | CCGGGAATTCAAAGCTAATAAGGCACTCGAGTGCCTTATTAGCTTTGAATTCTTTTTG |
| shRNA -2 | CCGGGGAATTCAAAGCTAATAAGGCCTCGAGGCCTTATTAGCTTTGAATTCCTTTTTG |
| shRNA -3 | CCGGGAAGGAATTCAAAGCTAATAACTCGAGTTATTAGCTTTGAATTCCTTCTTTTTG |

Supplementary table 2 The Crisper Cas9 gRNA sequences

| **Target genes** | **Sequences** | **Target location** |
| --- | --- | --- |
| Human *SNAIL* #1 | TGCGCTACTGCTGCGCGAAT | Exon 1 |
| Human *SNAIL* #2 | AGAGCGCGGCATAGTGGTCG | Exon 1 |
| Human *SNAIL* #3 | AGCATTGGCAGCGAGGCGGT | Exon 2 |
| Human *SNAIL* #4 | CCGCAGGTTCCGCAGACGCA | Exon 2 |

Supplementary table 3 Antibody for western blotting, RIP and immunohistochemistry

| **Antibody** | **Company** | **Cat No.** |
| --- | --- | --- |
| β-actin | YEASEN | 30101ES50 |
| Peroxidase-Conjugated Goat Anti-Rabbit IgG(H+L) | YEASEN | 33101ES60 |
| HRP-labeled Goat Anti-mouse IgG(H+L) | YEASEN | 33201ES60 |
| AGO2 | Abcam | ab32381 |
| IgG | Abcam | ab172730 |
| E-cadherin | Abcam | ab40772 |
| N-cadherin | Abcam | ab18203 |
| Vimentin | Abcam | ab92547 |
| Snail | ABclonal | A5243 |
| Alexa Flour 488 AffiniPure Goat Anti-Rabbit IgG (H+L) | YEASEN | 33106ES60 |
| Alexa Fluor 594 AffiniPure Goat Anti-Rabbit IgG (H+L) | YEASEN | 33112ES60 |

Supplementary table 4 The qRT-PCR primers used in this study

| **Gene** | **Forward primer (5’-3’)** | |  | **Reverse primer (5’-3’)** |
| --- | --- | --- | --- | --- |
| SWT1 | TGAAGACAACAGAAGTACCAGGT | |  | ACGATCTAGCTCTTGCATAACGA |
| SNAIL | CGAGTGGTTCTTCTGCGCTA | |  | CTGCTGGAAGGTAAACTCTGGA |
| β-actin | ACAGAGCCTCGCCTTTGCC | |  | GATATCATCATCCATGGTGAGCTGG |
| GAPDH | GGAGCGAGATCCCTCCAAAAT | |  | GGCTGTTGTCATACTTCTCATGG |
| U6 | CTCGCTTCGGCAGCACA | |  | AACGCTTCACGAATTTGCGT |
| **Convergent primer** | |  |  |  |
| hsa_circ_0004689 | CACAGTGACTAAAAAGCAGGAAGG | |  | ACCAGATTTCTTGATGCCTGC |
| **Divergent primer** |  | |  |  |
| hsa_circ_0111484 | GCTTTTGGAAACCTTTGGATGG | |  | ACTCTCCAGGTAAATCCACACT |
| hsa_circ_0015674 | CTAAAATGCTGTCTCCAGCACC | |  | TTTTGGTCTCCTTCTGAGAGTG |
| hsa_circ_0111492 | GCTTTTGGAAACCTTTGGATGG | |  | ACTCTCCAGGTAAATCCACACT |
| hsa_circ_0111468 | AGAGAGAAAAACCCCAGCAAGT | |  | GGTGATGAGGTGGTGGTGTC |
| hsa_circ_0015675 | TCCACAGACCTTTGCTCAAGT | |  | CCACACGTGCAGCATGAAG |
| hsa_circ_0111471 | GGACTGTTCATGAGTGGAAACG | |  | TAGGTCTTTGGGATGAAGAACCG |
| hsa_circ_0111475 | CTTTTGGAAACCTTTGGATGGAG | |  | GTCTTTGGGATGAAGAACCGAT |
| hsa_circ_0111470 | AGAGAGAAAAACCCCAGCAAGT | |  | GGTGATGAGGTGGTGGTGTC |
| hsa_circ_0111488 | AACCCAGCAGGCATCAAGAA | |  | ACAATTAGAAGCTTTCTGTCTGAAG |
| hsa_circ_0111490 | AGTTCTCATCTTCCCCAACCC | |  | TTTACTGAGTGACTTCACACCAC |
| hsa_circ_0111487 | TCCACAGACCTTTGCTCAAGT | |  | CAATTAGAAGCTTTCTGTCTGAAGT |
| hsa_circ_0141519 | AGACCTTTGCTCAAGTAAACAACC | |  | CCAAAATTGGGTGATGAGGTGG |
| hsa_circ_0111496 | ATGGCAGCTGTGAGGGATATT | |  | CTGCAAATGTCTGAAGGAGGTTG |
| hsa_circ_0015671 | AGTTAATGGCAGCTGTGAGGG | |  | GCATTTACTTCCTCCGAGGCT |
| hsa_circ_0111494 | AGTTAATGGCAGCTGTGAGGG | |  | TGCTCTCAAGCAGAATGGACAG |
| hsa_circ_0015676 | AAAATGCTGTCTCCAGCACC | |  | ACAATTAGAAGCTTTCTGTCTGAAG |
| hsa_circ_0111491 | TTAATGGCAGCTGTGAGGGAT | |  | CACACCACTTATTAGGCCTTTGT |
| hsa_circ_0111485 | TCCACAGACCTTTGCTCAAGT | |  | CCACACGTGCAGCATGAAG |
| hsa_circ_0015672 | AAACATTGGTTGGCTGTATTTGGAT | |  | GAGGTGGTGGTGTCTTTCCTC |
| hsa_circ_0015670 | ACCTGCAGTTCATTTCATCAACG | |  | TTCCTCCGAGGCTTGAACTC |
| hsa_circ_0111481 | CTAAAATGCTGTCTCCAGCACC | |  | GCATCTCTTGATCTGCATCCTG |
| hsa_circ_0111483 | TAAAATGCTGTCTCCAGCACCAG | |  | ACTCTCCAGGTAAATCCACACTT |
| hsa_circ_0111493 | AGTTCTCATCTTCCCCAACCC | |  | TAGCTCTCTTTCAAGGGTGTTGT |
| hsa_circ_0111474 | CTAAAATGCTGTCTCCAGCACC | |  | TTTGGTCTCCTTCTGAGAGTGT |
| hsa_circ_0111480 | TCAGTCAATACAACTTGCATCCC | |  | CATCTCTTGATCTGCATCCTGG |
| hsa_circ_0111489 | TCTTCCACAGACCTTTGCTC | |  | TTACTGAGTGACTTCACACCAC |
| hsa_circ_0015677 | GTTAATGGCAGCTGTGAGGG | |  | ACAATTAGAAGCTTTCTGTCTGAAG |
| hsa_circ_0111469 | GGACTGTTCATGAGTGGAAACG | |  | GTGATGAGGTGGTGGTGTCTT |
| hsa_circ_0111477 | CCCAGCAGGCATCAAGAAATC | |  | TAGGTCTTTGGGATGAAGAACCG |
| hsa_circ_0111486 | TAATGGCAGCTGTGAGGGATATT | |  | TGCAGCATGAAGCTCTTCTACTAT |
| hsa_circ_0111473 | CCTGCAGTTCATTTCATCAACG | |  | TTGGTCTCCTTCTGAGAGTGT |
| hsa_circ_0141518 | CGGACTGTTCATGAGTGGAAAC | |  | CCCACAGGATTCTTTGCTGG |
| hsa_circ_0111495 | AAAAGTTAATGGCAGCTGTGAGG | |  | CAGGAGTTCTGTCAGCACGG |
| hsa_circ_0111479 | GGAATTCAAAGGATTTTGGCCC | |  | TTGGTCTCCTTCTGAGAGTGTC |
| hsa_circ_0111472 | CTGCACGTGTGGGAAAAAGTG | |  | TTTGGTCTCCTTCTGAGAGTGTC |
| hsa_circ_0015673 | ACCTGCAGTTCATTTCATCAACG | |  | TTGGTCTCCTTCTGAGAGTGTC |
| hsa_circ_0111482 | AGTTAATGGCAGCTGTGAGGG | |  | TGCATCTCTTGATCTGCATCCT |
| hsa_circ_0111478 | ATGGCAGCTGTGAGGGATATT | |  | TAGGTCTTTGGGATGAAGAACCG |
| hsa_circ_0004689 | TGGCAGCTGTGAGGGATATT | |  | TGACCTTGTACTGAAAGCATGT |
| hsa_circ_0111497 | GCCCAGGAAATTTATGATTGTGT | |  | TTAGGAAGTTATAGAGGGTCTCAAC |
| hsa_circ_0111476 | TCTTCCACAGACCTTTGCTC | |  | TTGGTCTCCTTCTGAGAGTGT |
| hsa-miR-1208 | GCGCGTCACTGTTCAGACA | |  | AGTGCAGGGTCCGAGGTATT |
| hsa-miR-1225-3p | TGAGCCCCTGTGCCGC | |  | AGTGCAGGGTCCGAGGTATT |
| hsa-miR-1233-3p | CGCGTGAGCCCTGTCCTC | |  | AGTGCAGGGTCCGAGGTATT |
| hsa-miR-1251-5p | CGCGACTCTAGCTGCCAAA | |  | AGTGCAGGGTCCGAGGTATT |
| hsa-miR-1252-5p | GCGCGAGAAGGAAATTGAATT | |  | AGTGCAGGGTCCGAGGTATT |
| hsa-miR-1322 | CGCGGATGATGCTGCTG | |  | AGTGCAGGGTCCGAGGTATT |
| hsa-miR-215-5p | GCGCGATGACCTATGAATTG | |  | AGTGCAGGGTCCGAGGTATT |
| hsa-miR-192-5p | GCGCGCTGACCTATGAATTG | |  | AGTGCAGGGTCCGAGGTATT |
| hsa-miR-194-5p | CGCGTGTAACAGCAACTCCA | |  | AGTGCAGGGTCCGAGGTATT |
| hsa-miR-203a-3p | CGCGGTGAAATGTTTAGGAC | |  | AGTGCAGGGTCCGAGGTATT |
| hsa-miR-370-3p | GGCCTGCTGGGGTGGAA | |  | AGTGCAGGGTCCGAGGTATT |
| hsa-miR-431-5p | GCGTGTCTTGCAGGCCGT | |  | AGTGCAGGGTCCGAGGTATT |
| hsa-miR-495-3p | GCGAAACAAACATGGTGCA | |  | AGTGCAGGGTCCGAGGTATT |
| hsa-miR-526b-5p | GCGCTCTTGAGGGAAGCACT | |  | AGTGCAGGGTCCGAGGTATT |
| hsa-miR-526b-3p | CGCGGAAAGTGCTTCCTTTT | |  | AGTGCAGGGTCCGAGGTATT |
| hsa-miR-570-3p | CGCGCGAAAACAGCAATTAC | |  | AGTGCAGGGTCCGAGGTATT |
| hsa-miR-578 | GCGCGCTTCTTGTGCTCTAG | |  | AGTGCAGGGTCCGAGGTATT |
| hsa-miR-580-3p | GCGCGTTGAGAATGATGAATC | |  | AGTGCAGGGTCCGAGGTATT |
| hsa-miR-589-5p | GCGTGAGAACCACGTCTGC | |  | AGTGCAGGGTCCGAGGTATT |
| hsa-miR-607 | CGCGGTTCAAATCCAGATC | |  | AGTGCAGGGTCCGAGGTATT |
| hsa-miR-654-3p | CGCGTATGTCTGCTGACCAT | |  | AGTGCAGGGTCCGAGGTATT |
| hsa-miR-7-5P | CGCGTGGAAGACTAGTGATTTT | |  | AGTGCAGGGTCCGAGGTATT |
| hsa-miR-892a | CGCGCACTGTGTCCTTTCT | |  | AGTGCAGGGTCCGAGGTATT |

Supplementary table 5 The probe sequence for circSWT1 and miR-181a-5p

| **Probe** | **Sequence** |
| --- | --- |
| circSWT1 | TCAAAGCTAATAAGGCAGTGG |
| miR-370-3p | ACCAGGTTCCACCCCAGCAGGC |
