# Supplementary material for: Circular RNA hsa_circ_0004689 (circSWT1) promotes NSCLC progression via the miR‐370‐3p/SNAIL axis by inducing cell epithelial‐mesenchymal transition (EMT)
Source: Cancer Med. 2022 Dec 19;12(7):8289–305. doi: 10.1002/cam4.5527 (PMC10134258; doi:10.1002/cam4.5527)
Supplement: Supplementary file 3 — Appendix S1. [file CAM4-12-8289-s002.docx]

**Supplementary Materials and Methods**

***Western blotting***

Protein level was assessed by Western blotting. The lysate buffer was mixed with PMSF (Boster, China), and then, the mixture was incubated for 5 min at room temperature. Cells were collected and washed with PBS three times and then lysed on ice for 30 min. After the cells were centrifuged (12000 r/min, 4°C), the supernatant was collected and mixed with loading buffer (Beyotime, China). Then, the mixture was boiled for 10 min and allowed to cool at room temperature. Proteins with different molecular weights in the samples were separated by sodium dodecyl sulfate–polyacrylamide gel electrophoresis (SDS-PAGE), and large-molecular-weight proteins moved slowly and covered a short distance. The protein was transferred from the 10% SDS-PAGE gel (EpiZyme, China) to PVDF membranes (Millipore, Ireland). Next, PVDF membranes were removed and incubated with protein-free rapid blocking buffer for 10 to 15 min (EpiZyme, China). Then, we used TBST to wash the membrane three times and incubated it with the primary antibody overnight at 4°C. The next day, the PVDF membranes were incubated with the secondary antibody for 1 h at room temperature. Finally, an HRP enhanced chemiluminescence kit (Fdbio Science, China) was used to treat the PVDF membranes, and images were obtained using a Tanon Imaging System (Tanon, China). The primary antibodies were described as following: monoclonal rabbit anti-E Cadherin (dilution 1:1000, abcam, USA); polyclonal rabbit anti-N Cadherin (dilution 1:1000, abcam, USA); monoclonal rabbit anti-Vimentin (dilution 1:1000, abcam, USA); polyclonal rabbit anti-Snail (dilution 1:1000, Abclonal, China).

***Immunohistochemistry***

The level of the target protein was detected by IHC. Briefly, the obtained specimens were first made into paraffin sections, and then, they were baked in a constant-temperature drying oven (65℃, 30 min). Thus, the samples were sequentially deparaffinized and rehydrated by xylene, absolute ethanol, 95% ethanol, 85% ethanol and 75% ethanol. Next, the samples were repaired with Citrate Antigen Retrieval Solution (50×) or EDTA Antigen Retrieval Solution (50×) (Absin, China). Then, color development for immunohistochemical staining was performed with the SP-9000 SPlink Detection Kit (ZSGB-BIO, China) according to the instruction manual. First, H_2_O_2_ was added to the paraffin sections to block endogenous peroxidase for 10 min at room temperature. Next, goat serum was added to block for 30 min at room temperature. Then, the specific primary antibody was added to the samples overnight at 4℃. The next day, biotin-labeled goat anti-mouse/rabbit IgG polymer was added first, and then, the HRP-labeled streptavidin regent was incubated with the samples. After addition of a DAB diluent (ZSGB-BIO, China) to develop the color, the nucleus was stained with hematoxylin (Servicebio, China) for 2 to 5 min. Then, the samples were rinsed in hematoxylin differentiation solution (Servicebio, China) for 10 s, and hematoxylin bluing solution (Servicebio, China) was added to promote the bluing process. Next, the samples were washed in tap water for 2 min and dehydrated with alcohol. The results were observed by a microscope. The primary antibodies were described as following: monoclonal rabbit anti-E Cadherin (dilution 1:500, abcam, USA); polyclonal rabbit anti-N Cadherin (dilution 1:100, abcam, USA); monoclonal rabbit anti-Vimentin (dilution 1:500, abcam, USA); polyclonal rabbit anti-Snail (dilution 1:200, Abclonal, China).

**RNA isolation and qRT-PCR analysis**

For cell RNA isolation, first, cells were washed (PBS×2), digested, collected to EP tube, and then centrifuged (1500 r/min, 5 min). Then, 1 ml of TRIzol reagent (Invitrogen, USA) was added to the EP tube and incubated for 5 min at room temperature. Next, 200 μl of chloroform was added to EP tube. The mixture was shaken for 15 s, incubated for 2 min and then centrifuged (12000 g, 15 min, 6℃). The supernatant was transferred to another EP tube, mixed with 500 μl of isopropyl alcohol (Sigma-Aldrich, USA) and incubated at room temperature for 10 min. Next, the mixture was centrifuged (12000 g, 6℃, 10 min). We discarded the supernatant and washed the sample with 75% ethanol, and the new mixture was centrifuged (7500 g, 6℃) for 5 min. After removal of the supernatant, the precipitate was incubated at room temperature for 30 min, and 50 μl of RNase-free water (Solarbio, China) was added to dissolve the precipitate. Then, a NanoDrop 2000 (Thermo Fisher) was used to detected the concentration of total RNA. The isolated RNA was reverse transcribed into cDNA according to the instructions of the manufacturer’s kit (Yeasen, China). qRT-PCR was performed with SYBR Green Real-time PCR Master Mix (Yeasen). GAPDH was used as an internal control for circRNAs and mRNA, and U6 snRNA was used as a reference for miRNA. The 2^-ΔΔCt^ method was used to evaluate the level of RNA.
